# Evidence of lower oxygen reserves during labour in the growth restricted human foetus: a retrospective study

**DOI:** 10.1186/s12884-017-1392-7

**Published:** 2017-07-01

**Authors:** Silvia Parisi, Clara Monzeglio, Rossella Attini, Marilisa Biolcati, Bianca Masturzo, Manuela Mensa, Marina Mischinelli, Eleonora Pilloni, Tullia Todros

**Affiliations:** Department of Obstetrics and Gynaecology, Sant’Anna Hospital, University of Turin, Via Ventimiglia 3, Turin, Italy

**Keywords:** Cardiotocography, Labour, Small for gestational age, Foetal growth restriction, Hypoxia

## Abstract

**Background:**

The aim of the present study is to test the hypothesis that Growth Restricted foetuses (FGR) have the tendency to develop more pathological cardiotocograpic tracings during labour than do appropriate for gestational age foetuses and that there is a shorter time lapse from the beginning of labour and the advent of a pathological cardiotocograpic tracing.

**Methods:**

The study was carried out at the Maternal-Foetal Medicine Unit of the Sant’Anna University Hospital, Turin, Italy. A total of 930 foetuses born at term between January and December 2012 were analysed: 355 small for gestational age (SGA) comprising both constitutional small for gestational age and growth restricted foetuses (cases group) and 575 Appropriate for Gestational Age (AGA) foetuses (control group). Tracings were evaluated independently by two obstetric consultants, according to the International Federation of Gynaecology and Obstetrics (FIGO) classification. The main outcomes considered were the incidence of pathological cardiotocograpic tracings and the time interval between the beginning of labour and the advent of pathological cardiotocograpic tracing.

The Student’s t-test, chi-square test and ANOVA were used for comparisons between cases and controls and amongst groups. Significance was set at <0.05. Univariate and multivariate odds-ratios were calculated.

**Results:**

Foetuses with birthweight <3rd centile (growth restricted foetuses) more frequently presented pathological cardiotocograpic tracings in labour than did controls (43.8% vs. 21.6%; *p* < 0.001). Pathological cardiotocograpic tracing developed faster in the foetuses with birthweight <3rd centile group (53′, 0′-277′) than it did in the control group (170.5′, 0′-550′; *p* < 0.05).

A higher induction rate was observed in the cases (29.6%) than in the control group (17%), with statistical significance *p* < 0.001. To correct for this possible confounding factor a multivariate logistic regression analysis was performed. It confirmed a statistically significant increased risk of pathological cardiotocographic tracings in the FGR group (OR 1.63; CI 1.30–2.05).

**Conclusion:**

The results confirm the hypothesis that Growth Restricted foetuses (FGR) have fewer oxygen reserves to deal with labour. Our results underscore the importance of the prenatal detection of these foetuses and of their continuous cardiotocographic monitoring during labour.

**Electronic supplementary material:**

The online version of this article (doi:10.1186/s12884-017-1392-7) contains supplementary material, which is available to authorized users.

## Background

Currently, cardiotocography (CTG) is the method adopted to detect foetal hypoxia during labour to avoid tissue damage. Indeed, labour is a stressful event for the foetus as the uterine contractions reduce uteroplacental blood flow and increase intramniotic and foetal pressure [1, 2]. Moreover, the foetus is subjected to acid-base balance changes in the maternal blood with a first phase of respiratory alkalosis, due to maternal hyperventilation and a subsequent compensatory metabolic acidosis [3]. Under physiological conditions, the foetus has adequate oxygen reserves to survive labour without injury. However, it is generally maintained that if the foetus is already hypoxic when the first stage of labour starts, there is a higher risk of asphyxia. Animal studies show that foetuses with pre-existing stable hypoxemia during transient reduction in the uterine blood flow, mimicking uterine contractions, had progressive metabolic acidosis and a quicker and higher decrease in foetal heart rate (FHR), than did normoxic animals [4, 5].

Chronic foetal hypoxia during human pregnancy may cause restriction of foetal growth. Foetal Growth Restriction (FGR) is usually considered a risk factor for acidosis and asphyxia during labour. Therefore, guidelines suggest continuous electronic foetal monitoring (EFM) be used throughout labour in these cases [6–9]. However, to the best of our knowledge there are no clinical studies showing how frequently CTG becomes pathological during labour or the pattern and speed of deterioration of CTG in FGR at term.

This study aimed at: determining whether FGR leads to the foetus having fewer oxygen reserves and being more prone to developing pathological CTG tracings during labour than Appropriate for Gestational Age (AGA) foetuses and evidencing any differences in the time-lapse from the start of labour to a pathological CTG tracing in FGR and AGA.

## Methods

This retrospective study was carried out at the Maternal-Foetal Medicine Unit of Sant’Anna University Hospital, Turin, Italy. A total of 930 foetuses born at term between January and December 2012, 355 Small for Gestational Age (SGA) (cases group) and 575 AGA foetuses (control group), were analysed. The inclusion criteria were: single pregnancy, term delivery, labour, cardiotocographic (CTG) recordings throughout labour. Moreover, we included in this study only women who were in hospital since the beginning of labour and in whom the beginning of labour was clearly determined and documented. The exclusion criteria were twin pregnancy, the absence of CTG tracing during the last 2 h before delivery, elective caesarean section, preterm delivery and uncertainty about the beginning of active phase of labour.

Term is defined as the period between 37 + 0 and 41 + 6 weeks of gestational age. In most cases, gestational age was based on the last menstrual period and first-trimester ultrasound scan. If ultrasound dating were discordant with the last menstrual period by seven days or more, then only ultrasound dating was used to calculate gestational age. AGA describes newborns ≥10th ≤ 90th percentile for gender and gestational age according to Italian charts [10]. SGA defines newborns with a weight of less than the 10th percentile for gestational age and gender [10].

There was no data on foetal growth or uterine and/or umbilical artery Doppler in most of our SGA cases. Therefore, a lower birthweight cut-off (3rd centile) was used to identify FGR [11, 12]. Consequently, the cases group was divided into two sub-groups:

1. those with a birthweight of < the 3rd percentile (96 cases, FGR);
2. those with a birthweight between the 3rd and 10th percentile (259 cases, likely comprising both FGR and “constitutional” SGA).

CTG recordings of the last 2 h before delivery were taken into consideration in all cases. The tracings were evaluated blindly by two independent obstetric consultants. FHR patterns were classified as normal, suspicious, pathological and preterminal, according to the FIGO classification [13]. A pathological pattern is defined as having a baseline heart rate of >170 beats/min or between 150 and 170 beats/min with an amplitude of variability of <five beats/min, bradycardia <100 beats/min for more than 3 min, a persistence of heart rate variability of < five beats/min for >60 min, late decelerations or complicated variable decelerations with a duration of >60 s, a sinusoidal pattern [13].

In cases with pathological CTG, we analyzed the whole tracing from the beginning of labour.

The main outcomes considered were a pathological CTG and the time interval between the beginning of labour and a pathological CTG. The beginning of the active phase of labour was diagnosed if the cervix was effaced in a nulliparous woman and dilated by a minimum of 3 cm with regular uterine contractions at least every 5 min, lasting no less than 20 s.

The data were obtained retrospectively and included: the gestational age at delivery, mode of delivery, minutes of labour, the weight of the neonate, the Apgar score at 5 min, umbilical artery lactate values and any need for neonatal resuscitation.

Adverse outcomes included: a 5 min Apgar score of < seven, lactate values suggestive of acidosis (umbilical artery lactate values of > 7 mmol/L) and/or the necessity of neonatal resuscitation [14, 15].

As some authors reported a higher rate of adverse neonatal outcomes in male foetuses our data were also analysed separately for male and female foetuses [16, 17]. Descriptive analysis was performed as appropriate (average and standard deviation for parametric and median and range for non-parametric data). The Student’s t-test, chi-square test and ANOVA were used for comparisons between cases and controls and amongst groups. Significance was set at <0.05. Univariate and multivariate odds-ratios were performed as implemented on SPSS vers. 18.0 for Windows (SPSS, Chicago IL, USA).

## Results

The gestational age at birth was similar in all groups. More pathological CTG in labour were observed in the whole cases group than in controls (39.1% vs. 21.6%; *p* < 0.001). There was a statistically significant difference in the pathological CTG rate between both the sub-groups (birthweight <3rd centile and birthweight ≥3rd < 10th centile) and controls, but not between the two sub-groups themselves (Table 1). Moreover, there was a shorter-time lapse between the beginning of labour and the pathological CTG in the whole of the cases group than in controls. There was a constant decrease in the time-lapse starting from the control group (median 170.5′) to an intermediate level in the birthweight sub-group ≥3rd < 10th centile (median 130′) and the shortest time-lapse was observed in the birthweight <3rd centile (median 53′, *p* < 0.05). When the two sub-groups were compared (birthweight < 3rd centile and birthweight ≥3rd < 10th centile) a statistically significant difference in the time-lapse was observed (Table 1). Whilst, there was no difference in time-lapses from the onset of a pathological CTG and delivery in any of the groups. Labour was significantly shorter in the cases group and in each of the sub-groups (birthweight < 3rd centile and birthweight ≥3rd < 10th centile) compared to controls and was significantly shorter in birthweight <3rd centile than in birthweight ≥3rd <10th centile (Table 1), during both the first and second stage of labour.

**Table 1:** Main outcomes

	Birthweight <3rd centile – p1 (96 cases)	Birthweight ≥ 3rd < 10^th^centile – p2 (259 cases)	All Cases – p3 (355 cases)	Controls – p4 (575 cases)	*p* value
Pathological CTG	42 (43.8%)	97 (37.4%)	139 (39.1%)	124 (21.6%)	p1 vs p2 = NS p1, p2 and p3 vs p4 < 0.001
Patterns more frequently observed in pathological CTG	Severe variable/late decelerations 36/42 (85.7%)	Severe variable/late decelerations 79/97 (81.4%)	Severe variable/late decelerations 115/139 (82.7%)	Severe variable/late decelerations 108/124 (87.1%)	NS
Minutes from beginning of labour and pathological CTG (median, range)	53 (0–277)	130 (0–555)	118 (0–555)	170.5 (0–550)	p1 vs p2 and p4, p2 vs p4 < 0.05 p3 vs p4 < 0.001
Minutes from pathological CTG and delivery (median, range)	67.5 (13–200)	55 (8–200)	59 (8–200)	50 (7–306)	NS
Minutes of labour (median, range)	136.5 (40–603)	172 (5–635)	160 (5–635)	200 (23–840)	p1 vs p2 < 0.05 p1, p2 and p3 vs p4 < 0.001

There was no statistical difference in any of the groups for Caesarean sections or operative delivery (vacuum extractor) rate (Table 2). A 5 min Apgar score of < seven was observed in 3/96 cases (3.1%) in the birthweight sub-group <3rd centile and in 2/575 cases (0.3%) in the control group (*p* < 0.05). Although no cases had an Apgar score of < seven at 5 min in the birthweight sub-group ≥3rd < 10th centile, there was a neonatal resuscitation rate of 3.5% in the latter, vs. 1% in the control group (*p* < 0.05). No difference in the rate of abnormal lactate was observed (Table 2).

**Table 2:** Neonatal outcomes

	Birthweight <3rd centile – p1 (96 cases)	Birthweight ≥3rd < 10^th^centile – p2 (259 cases)	All Cases – p3 (355 cases)	Controls – p4 (575 cases)	*p* value
Vaginal delivery	74 (77.1%)	207 (79.9%)	281 (79.1%)	463 (80.6%)	NS
Caesarean section	16 (16.7%)	33 (12.7%)	49 (13.8%)	73 (12.7%)	NS
Vacuum extractor	6 (6.2%)	19 (7.4%)	25 (7%)	38 (6.7%)	NS
Neonatal weight (media ± DS)	2435.9 ± 268	2664 ± 142	2587.6 ± 221	3456.4 ± 327.4	p1 vs p2, p1, p2 and p3 vs p4 < 0.001
Apgar < 7 at 5′	3 (3.1%)	0	3 (0.8%)	2 (0.3%)	p1 vs p2 and p4 < 0.05 p2 and p3 vs p4 = NS
Lactate suggestive for acidosis	2/52 (3.8%)	4/125 (3.2%)	6/177 (3.3%)	10/214 (4.7%)	NS
Neonatal resuscitation	2 (2.1%)	9 (3.5%)	11 (3.1%)	6 (1%)	p1 vs p2 and p4 = NS p4 vs p2 and p3 < 0.05

There were 75% undiagnosed FGR in the birthweight sub-group <3rd centile and 92.7% in the birthweight sub-group ≥3rd < 10th centile, with a statistically significant difference (*p* < 0.001). There were no statistical differences in any of the outcomes when foetuses were considered according to gender. A higher induction rate was observed in the cases group (29.6%) and in each of the sub-groups (<3^rd^centile: 34.4%; ≥ 3rd < 10th centile: 27.8%), than in the control group (17%), with statistical significance (*p* < 0.001). To correct for this possible confounding factor a multivariate logistic regression analysis was performed (Table 3). It confirmed a statistically significant increased risk of pathological CTG in the cases group and subgroups (Table 3).

**Table 3:** Odds of developing a pathological CTG in the different groups

	Induction	Univariate Odds Ratio (95% Confidence Interval)	Multivariate Odds Ratio (95% Confidence Interval)
Controls	17%	1	1
All Cases	29.6	2.34 (1.75–3.13)	2.20 (1.64–2.96)
Birthweight ≥3rd < 10^th^centile	27.8%	2.18 (1.58–3.00)	2.06 (1.49–2.85)
Birthweight <3rd centile	34.4%	2.83 (1.80–4.43)	1.63 (1.30–2.05)

## Discussion

To date the literature on the CTG monitoring of FGR foetuses in labour is scant.

Our results confirm the hypothesis that FGR are more prone to pathological CTG in labour than controls and that the time-lapse between the beginning of labour and the occurrence of pathological CTG is shorter in cases than controls.

These results are in agreement with animal studies reporting that chronically hypoxic foetuses had a lower resistance to labour stress than did normoxic foetuses. Indeed, Itskovitz et al. observed that chronically hypoxic lambs had a quicker and higher decrease in pO_2_ levels and a delayed deceleration of the FHR, after a transitory reduction in uterine blood flow of 10/20 s, similar to what is observed during a uterine contraction during labour [4]. Westgate et al. investigated into the role a transitory occlusion of the umbilical cord in spontaneous hypoxic sheep plays in the development of progressive metabolic acidosis, FHR changes and an abnormal electrocardiogram (ECG). They concluded that chronic hypoxic sheep had more of these abnormalities than did normoxic sheep [5].

Human SGA foetuses are a heterogeneous group which includes both foetuses that are small, but otherwise normal and foetuses that have suffered growth restriction due to chronic hypoxia and under nutrition. The latter are the ones whose conditions are expected to rapidly deteriorate during labour. The differential diagnosis between the two groups is usually based on the intrauterine growth curve and the uterine and/or umbilical artery Doppler. According to the most recent literature reports, any SGA with a centile of <3rd should be classified as FGR, whilst the SGA with a centile between ≥3rd and <10th would make up a heterogeneous group that includes both FGR and constitutional SGA [11, 12]. Given the retrospective design of our study, we could only rely on birthweight to distinguish between constitutional SGA and FGR. Our data confirm this subdivision based on birthweight. Indeed, foetuses with a centile of <3rd tended to have a higher incidence of pathological CTG, than did the foetuses with birthweight ≥3rd < 10th centile foetuses. Moreover, there was a shorter time-lapse between the start of labour and the onset of a pathological CTG in foetuses with birthweight <3rd centile, with a statistically significant difference from those observed in the ≥3rd < 10th centile foetuses.

The number of pathological CTG were surprisingly less than might have been expected, above all in the birthweigth <3rd centile foetuses. However, these findings may well be influenced by the fact that our data cover full term pregnancies (≥37 < 42 weeks) that went into labour and not those that had a preterm delivery and/or Caesarean section without labour. CTG tracings from each single group were analysed to evidence any typical patterns. However, all groups had the same most frequent finding i.e. severe variable or late deceleration, without any statistical significance.

Despite the higher rate of pathological CTG in our cases group, there was no increased incidence of Caesarean section or operative deliveries in this group.

This may be attributed to the shorter statistically significant labour we observed in the cases group, both those with a pathological CTG and normal CTG, compared to the AGA group. This leaves us with the question of how we might explain this finding. We propose two possible explanations:

1. the first one involves their reduced dimensions i.e. as the FGR are smaller, their labour course both for dilatation and expulsion, is briefer;
2. some animal and clinical studies have reported an up-regulation of the foetal hypothalamus-pituitary-adrenal axis in intrauterine growth restriction: with an increase in the production of glucocorticoids [18–22]. This seems to lead to a stimulation in the production of prostaglandins and, therefore, an increase in uterine contraction [22–24].

Moreover, in line with our findings, the main indication for a Caesarean Section in the AGA group was dystocia (63% of cases), whilst it was pathological CTG in the cases group (birthweight <3rd centile 87.6% of cases; birthweight ≥3rd <10th centile 60.6% of cases).

The neonatal outcomes in the birthweight <3rd centile were as expected i.e. there was a statistically significant increase in the 5 min Apgar score of < seven compared to the controls (3.1% vs. 0.3%; *p* < 0.05). The absence of any statistical difference between the lactates suggestive for acidosis in all three groups implies that the medical intervention was timely and efficacious. However, these results must be interpreted with caution because of the small number of cases with abnormal outcomes.

Although, as aforementioned, there was no statistically significant difference in adverse outcomes according to gender, there was a higher rate of pathological CTG’s in male foetuses in the birthweight sub-group < 3rd centile and in the control group and more Caesarean sections in both sub-groups and controls. Our data confirm the results of Simchen et al. that report a higher rate of Caesarean sections and instrumental deliveries in male term SGA and AGA foetuses than in female foetuses [16]. They related these findings to the differences in birthweight, especially in AGA foetuses. Our results lead us to speculate that the higher rate of pathological CTG in male foetuses plays a role in this question. Kwon et al. suggest that male SGA foetuses have an immature cardiovascular autonomic control system, an observation that might explain the increased rate of pathological CTG [17].

In our study, there was also a high rate of undiagnosed SGA (75% in the group with birthweight <3rd centile and 92.7% in the group with birthweight ≥3rd < 10th centile). This is higher than the reported rate of 50% to 60% [25, 26]. However, these findings may well be influenced by the fact that our data cover full term pregnancies (≥37 < 42 weeks) that went into labour and not those that had a preterm delivery and/or Cesarean section without labour. To the best of our knowledge this is the first clinical study analysing how frequently CTG becomes pathological during labour or the pattern and speed of deterioration of CTG in FGR at term. The most important limitation is the retrospective design of the study. In fact been retrospective, it isn’t possible to clearly distinguish true FGR from constitutional SGA. Another limitation is the relatively small number of cases included in the study. As our present study was retrospective no definitive conclusions may be drawn and further prospective studies are warranted.

## Conclusions

Our results confirm the hypothesis that FGR have low oxygen reserves to deal with labour. Moreover, our results underscore the importance of the prenatal detection of these foetuses and of their continuous CTG monitoring during labour. However, further prospective studies are warranted.

## Additional file

Additional file 1: “Cases”, “Birthweight <3 centile”, “Birthweight >3 < 10”, “Control group”. the data include all variables analysed in our study in the both cases and control groups. (XLS 236 kb)

## Abbreviations

AGA: Appropriate for gestational age
CTG: Cardiotocography
ECG: Electrocardiogram
EFM: Electronic foetal monitoring
FGR: Foetal growth restriction
FHR: Foetal heart rate
FIGO: International Federation of Gynaecology and Obstetrics
SGA: Small for gestational age
